# Facilitators of and barriers to participation in Long COVID research: A qualitative analysis

**DOI:** 10.1371/journal.pone.0346007

**Published:** 2026-05-06

**Authors:** Lily Chu, Theodore Lucas Hollar, Nancy Klimas, Jeanne Bertolli, Ana Lia Tamariz, Aryan Lajevardi, Ilya Pavlov, Ana Palacio

**Affiliations:** 1 Institute for Neuro-Immune Medicine, Nova Southeastern University, Fort Lauderdale, Florida, United States of America; 2 Dr. Kiran C. Patel School of Osteopathic Medicine, Nova Southeastern University, Fort Lauderdale, Florida, United States of America; 3 United States Centers for Disease Control and Prevention, Atlanta, Georgia, United States of America; 4 Department of Medical Education, Miller School of Medicine, University of Miami, Miami, Florida, United States of America; 5 Geriatric Research Education and Clinical Center, Miami VA Healthcare System, Miami, Florida, United States of America; 6 Department of Public Health Sciences, University of Miami, Miami, Florida, United States of America; Touro University California College of Pharmacy, UNITED STATES OF AMERICA

## Abstract

**Background:**

Meeting recruitment targets in an expeditious manner is essential to the successful completion of any research project. However, despite the high prevalence and debilitating nature of Long COVID (LC), recruitment of participants into some LC studies has been challenging.

**Objective:**

We aimed to a) identify factors influencing participation in LC research among individuals who were infected by SARS-CoV-2 but had declined participation in a LC study and b) to compare these factors to those previously recognized.

**Methods:**

Using a semi-structured guide, we interviewed thirteen people about their thoughts, experiences, and attitudes concerning participation in LC research. We imported interview transcripts into Nvivo 14 and analyzed them using thematic analysis. For coding, we used Charmaz’s coding scheme of open and focused coding within an application of the constant comparative method. Basic descriptive statistics were also deployed to supplement our qualitative analysis.

**Results:**

Fifteen factors describe the facilitators and barriers mentioned by participants. The top three facilitators were *Personal and social motivation, Incentives,* and *Familiarity and credibility of institutions involved with COVID-19*; the top three barriers were *Invasiveness, Social and political context,* and *Lack of time*. Skepticism and infringement on participants’ daily lives served as major obstacles to participation while trust, personal factors, and administrative factors encouraged participation. The facilitators and barriers identified are similar to those recognized previously except that in the politically charged atmosphere surrounding the COVID-19 pandemic, trust was especially vital.

**Conclusions:**

Many factors affect people’s decisions to participate in LC research but only some are modifiable by researchers. Building trust, offering incentives participants value, and removing logistical barriers may improve recruitment rates.

## Introduction

Many people continue to be sick for months to years following SARS-CoV-2 infection regardless of the severity of their acute illness. This persistence or recurrence of previous symptoms and/or appearance of new symptoms has been named Long COVID [1]. Long COVID encompasses a diversity of symptoms, has been linked to damage of multiple organ systems, and, as of September 2024, per a survey by the United States Census Bureau, affected up to 5.3% of the US adult population [1,2]. Approximately eighty percent of respondents with Long COVID reported that their health restricted their daily function with about 25% characterizing their limits as significant. In August 2022, 1.8 to 4 million Americans were estimated to be out of work due to Long COVID [3].

There are still many unknowns regarding COVID-19. It is not clear why some people experience a long-term illness after the initial phase of SARS-CoV-2 infection while others recover completely. Viral persistence, hypercoagulopathy, immune dysregulation, autoimmunity, and hyperinflammation are some of the mechanisms that have been suggested to instigate or perpetuate LC [4]. Multiple health organizations have proposed different ways to define Long COVID [5,6]. There are no diagnostic tests specific to Long COVID and currently, no United States Food and Drug Administration-approved treatments exist for it [5]. While progress has been made in understanding Long COVID [7] and many studies are underway [8], important questions about Long COVID remain [9]. Intensive research efforts may provide solutions, but research involves myriad steps.

Timely, adequate recruitment of study participants is essential to the successful completion of any research project. Despite Long COVID’s high prevalence and debilitating nature, recruitment of participants into some studies has been difficult. After almost two years of effort to sign people up, the United States’ National Institute of Health’s national Long COVID initiative, RECOVER, had met only 80% of their goal for adult study participants and less than 50% of the target for pediatric participants [10]. Our epidemiological study COVID-UPP (COVID-19: Understanding the Post-Viral Phase) faced similar challenges.

Low and slow recruitment rates are common across studies of different medical conditions. Up to 80% of clinical trials fail to meet enrollment targets or deadlines while 11% do not enroll a single study participant [11]. Many studies have explored reasons for recruitment challenges [12,13]. In a systematic review of discontinued trials, Briel et al. [12] classified recruitment barriers into five distinct categories: funding-related, research environment-related, design-related, trial team/ recruiter-related, and participant-related. In this paper we will focus on participant-related factors.

Early in the pandemic, Barre et al. [14] examined barriers and facilitators to participation in COVID-19 studies among Black individuals living in the United States. While the majority recognized the value of research, limited awareness of ongoing research, low health literacy, and mistrust of medical/scientific authorities were cited as obstacles. Barre thus recommended using trusted sources and familiar social media channels to educate this community about research opportunities. In contrast, to the best of our knowledge, there are currently no data available on the barriers and facilitators relevant to Long COVID study participation.

Using semi-structured interviews, we aimed to identify factors influencing participation in Long COVID research among individuals who have a documented history of SARS-CoV-2 infection and to compare these factors to those previously recognized in studies involving other medical conditions. We hope to suggest practical steps that can improve Long COVID study recruitment.

## Methods

### Study design

We conducted semi-structured interviews of adults residing in South Florida who had previously been infected by SARS-CoV-2 but were not hospitalized for their infection. The goal was to understand facilitators and barriers to enrollment in Long COVID research. This activity was reviewed by United States Centers for Disease Control and Prevention (CDC) and was conducted consistent with applicable federal law and CDC policy. The Nova Southeastern University Institutional Review Board reviewed the study protocol (2022−533) and determined it qualified for exempt status under Category 2, research involving interviews, for which written consent is generally not required. Participants were given the opportunity to review information about the study, examine materials concerning consent, and express willingness to participate three times: during initial contact with research staff, through written communication, and immediately before the interviews started. Oral consent was recorded on Zoom.

### Reflexivity and Integrity

Authors Lily Chu (LCh), Ana Palacio (AP), and Ana Lia Tamariz (AT) conducted the interviews. LCh and author T. Lucas Hollar (LH) created codes and classified segments of text under those codes.

LCh is a female medical researcher and board member for a non-profit scientific organization focused on myalgic encephalomyelitis/ chronic fatigue syndrome and related conditions like Long COVID. She has a background in internal medicine and possesses a Master of Science degree in health services research. She primarily uses quantitative research methods although she has published two mixed-methods papers focused on post-exertional symptoms.

LH is a male public health professor and researcher. He is trained in public administration, public policy, and organization theory. His research and evaluation experiences include quantitative and qualitative methods for investigating policy, systems, and environmental change initiatives aimed at improving population health, as well studying the social and political determinants of health.

AP, MD, MPH is a female professor of clinical medicine and a clinician investigator at the University of Miami. She was trained as a quantitative researcher but has over a decade of experience conducting qualitative studies including key informant interviews and program evaluation. She is also a provider in the Miami Veterans Administration (VA) Long COVID clinic.

AT is a female research assistant with a Master of Arts degree in art politics who currently works at the University of Miami. She met with co-authors AP and LCh several times to learn how to conduct in-depth interviews.

Prior to this project, none of the study participants knew or were known to LCh, LH, and AT. AP had served as the attending physician for some potential participants. During interviews, participants were informed that LCh, AP, and AT were members of the research team but not their titles or professional backgrounds.

Reflexivity involves qualitative researchers recognizing that their training, experiences, personal characteristics, beliefs, and biases will influence data collection, analysis, and interpretation. Since one can never truly isolate oneself from one’s research [15,16], as they analyzed the transcripts, LCh and LH engaged in epoche [17] or bracketing. To address reliability and control for biases and preconceptions, the research team held meetings every two to three weeks or as needed to review interpretations and analytical decisions. The analytic technique of constant comparison among codes and between interviews supported the consistency and dependability of the team’s analysis.

### Sampling and recruitment

Convenience sampling was used. Study participants were those who had been invited but decided not to participate in a 3-year longitudinal study, COVID-UPP. COVID-UPP was devised to track and compare symptoms and physical function among people aged 18−65 years old with documented evidence of SARS-CoV-2 infection from 6 area healthcare organizations in the state of Florida (Baptist Health, Broward Health, Jackson Health System, Nova Southeastern University clinics, UHealth and Miami VA Healthcare System). COVID-UPP assessments include online questionnaires every 3 months, an in-person interview and physical examination by research staff, bloodwork, spirometry, and echocardiography. Starting in January 2022, potential participants received an email from their healthcare system inviting them to complete an online survey if they were interested in taking part in COVID-UPP. Some individuals finished and submitted the survey while others did not. We wanted to understand this latter group’s decision-making process.

Beginning on December 13, 2022, we reached out to all individuals who either started but did not complete the COVID-UPP survey or received the email invitation, never clicked on the survey link, but demonstrated interest after their physician described this study. Consequently, our study population consists of individuals who had declined involvement in COVID-UPP. Besides documentation of SARS-CoV-2 infection within at least the last three months and rejection of participation in COVID-UPP, the only other requirement was a willingness to speak to us about the decision to not participate in COVID-UPP. Research staff contacted these individuals again by e-mail and then by telephone inviting them to participate in this study. We offered a $20 USD Amazon gift card as an incentive. Recruitment ended on March 23, 2023.

If they accepted the invitation, staff contacted them to explain study details, answer any questions or concerns, obtain consent, and arrange for a one-on-one interview using the video conference platform Zoom at a date and time chosen by the participant. Written information about the study and a consent form were sent to each participant three days prior to the interview. At the start of each meeting, interviewers asked study participants again if they had any concerns and confirmed their willingness to be interviewed. Participants were also reminded they could stop taking part at any time and did not have to answer any question. Oral consent was recorded on Zoom. Since AP and AT are both native Spanish speakers, participants could choose to be interviewed in Spanish.

### Data collection

We created a 14-item semi-structured interview (S1 File) based on literature about study recruitment, the authors’ professional experiences, and discussion among research team members. The initial interview guide was piloted on two interviewees; minor adjustments were made before using it for the remaining interviewees. Topics included the impact of COVID-19 on the person’s life; experiences obtaining medical care for Long COVID; prior engagement with other studies; opinions about Long COVID research; facilitators of and barriers to participation; trust in medical/scientific professionals and organizations; and preferred methods of contact.

From December 2022 until April 2023, three research team members (LCh, AP, AT) interviewed study participants using Zoom. Thus, participants could choose where they were interviewed. An additional staffer who had scheduled the meetings and was responsible for handling any technical issues was also present. Interviews were scheduled for 30 minutes but could be extended for up to an hour if participants wished. Participants could choose to turn off their cameras if they desired although interviewers kept their cameras on. Each participant was interviewed only once. Notes were not taken during or after the interviews.

Zoom audio auto-transcription was enabled. Research staff converted the transcripts into Microsoft Word or text documents. Transcripts were not shared with study participants but were verified for accuracy by staff listening to the video recordings of the interviews. The transcripts were then uploaded into Nvivo 14 for coding and analysis.

### Qualitative data analysis

We conducted an inductive analysis of the interview transcripts using thematic analysis [18]. For coding, we used Charmaz’s inductive coding scheme of open and focused coding [19] within an application of the constant comparative method. During open coding, we went line-by-line through the data with no preconceived coding protocol. The second phase was focused coding. Focused coding combines Glaser’s theoretical and selective coding procedures [20]. It clarifies categories within the data and breaks down categories within the data by examining all the data that each category emerging from the interviews covers and the variations in the data from each category. This allowed us to discover relationships between categories in ways that helped us explain the issues and events being studied, outlining “a framework that preserves the complexities of everyday life” [19]. We classified segments of transcripts under two types of codes: focused codes, summarizing thematic concepts within the topics of the interviewees’ conversations, and sentiment codes, classifying the topic under discussion as a barrier, facilitator, neither barrier nor facilitator, or neutral with regards to study participation.

The constant comparative method involves four stages [19]. First, as we coded the data, we constantly compared incidents applicable to each of the categories that emerged. Second, we integrated categories and their properties by way of comparing them to each other. Third, this comparison allowed us to delimit the themes that emerged. This delimiting process allowed us to achieve parsimony of the emerging variables and a sense of scope concerning the applicability of an emerging theoretical framework to a wide range of situations. Fourth, we were able to construct a theoretical framework for understanding and explaining the phenomenon described by the interviewees.

Authors LH and LC first read through all the transcripts to obtain an initial impression of the content. Next, using the same five transcripts, they each independently developed open codes and a code book. LH and LC met to discuss the open codes, examples of interview statements illustrating the open codes, how each open code was defined, and what was excluded from each open code. Another three transcripts were then selected to be coded separately using the agreed-upon open codes.

Based on the analysis of this subgroup of interviews, open codes were then refined and grouped into focused codes. Using these focused codes, all interviews were independently re-coded by each person. NVivo’s Kappa statistic was then used to compare the coding of each interview. When a focused code’s Kappa statistic fell below 0.4, denoting a moderate degree of disagreement between coders, sections of text corresponding to that focused code were examined synchronously by both researchers to understand why a discrepancy occurred. The definition of the focused code was reviewed, and the coders explained their thoughts to each other until agreement was reached. LH and LC also periodically met with the other members of the research team to review interpretations and analytical decisions associated with the coding process.

Saturation is defined as “the point at which gathering more data about a theoretical construct reveals no new properties, nor yields any further theoretical insights” [21]. In qualitative research, this threshold is not determined a priori but is observed during data collection and analysis.

### Frequency analyses of data

To supplement our qualitative analysis and to explore whether there were specific sub themes that were mentioned infrequently by many people or mentioned frequently by only a few people, we created a scatterplot of the number of text segments versus number of respondents. Next, for each category, we evaluated how many and what percentage of text segments corresponded to each sentiment. Then, we examined which categories were most common for each sentiment. The number and percentage of interviewees mentioning each subtheme and the context in which they were mentioned (as facilitator, barrier, or neutral factor) were also calculated.

Based on their responses to a question about whether their SARS-CoV-2 infections still affected their lives at the time of the interview, participants were classified as either “recovered” or “unrecovered.” The subthemes identified as the top facilitators and barriers by each group were then compared.

Results of analyses were not shared with study participants before submission for publication.

## Results

### Demographics

Ninety-eight individuals entered their e-mail address on the invitation form but did not complete it. These 98 were contacted by staff and 13 (13%) agreed to be interviewed. The median age of interviewees was 52 (range 25−65), 46% were female, and 31% self-identified as African American, 38% as Hispanic and 31% were Non-Hispanic White. Two participants chose to be interviewed in Spanish. Seven interviewees considered themselves “recovered” while five interviewees stated they were still sick, that is, “unrecovered” after having COVID-19. One interviewee could not be classified as they stated they were “recovered” but proceeded to describe continuing symptoms. Participants declined participation in COVID-UPP at different stages of the invitation process: two merely offered their names and e-mail on the invitation survey; two started but did not complete the invitation survey; six agreed to answer questionnaires as part of COVID-UPP but deferred in-person visits and testing; and three did not click on the invitation survey but agreed to be interviewed for this study after encouragement from their physician.

### Themes, subthemes, and constituting factors

**Fig 1:**
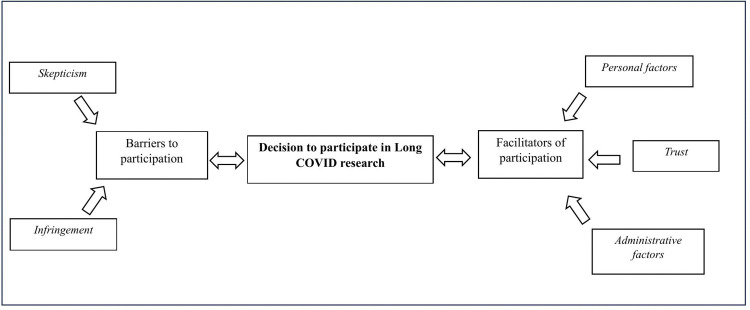
Major themes associated with barriers to and facilitators of participation in Long COVID research.

**Fig 2:**
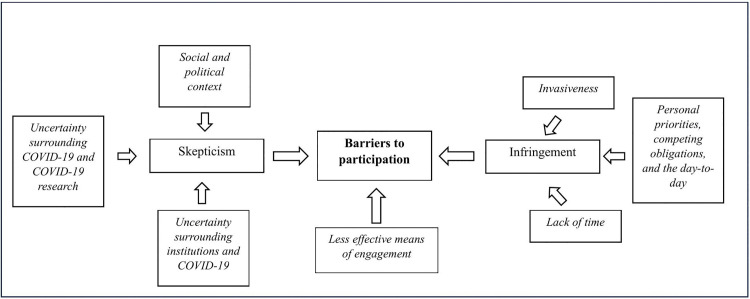
Themes and subthemes associated with barriers to participation in Long COVID research. Skepticism concerning COVID-19, less effective means of engagement, and perceived infringement on individual participant’s lives served as barriers to participation.

**Fig 3:**
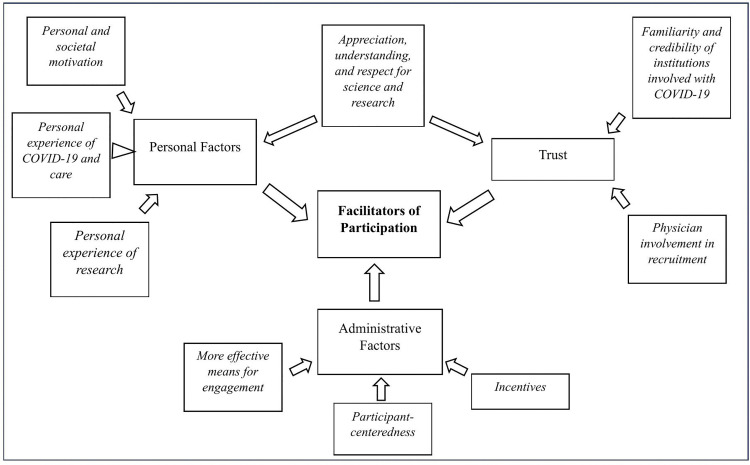
Themes and subthemes associated with facilitators of participation in Long COVID research. Trust, administrative factors, and personal factors play into individuals’ willingness to participate in Long COVID research.

Based on the interviews, we derived themes impeding and facilitating participation as well as an overarching theme (S1-S3 Tables) that helped us understand what the interviewees were expressing within their contexts. Saturation occurred during the first ten interviews, wherein no additional interview text contributed to the creation of new categories or themes. Fig 1 illustrates our overall unifying model while Figures 2 and 3 show the themes and subthemes constituting facilitating and obstructing factors. Skepticism and infringement experienced by individuals discouraged participation while trust, administrative factors, and personal factors supported individuals’ willingness to participate. Below, we summarize the ideas expressed in each theme and subtheme and provide selected quotes exemplifying the subtheme. Information in parentheses following each quote identify individual interviewees by a number, their age, and their self-assessed recovery status.

### Skepticism

Skepticism can diminish individuals’ willingness, comfort, confidence, and sense of safety in participating. Skepticism subthemes were *Social and political context, Uncertainty surrounding institutions and COVID-19,* and *Uncertainty surrounding COVID-19 and COVID-19 research.*

*Social and political context* captures interviewees’ expressions of how they perceived others’ and their own political and cultural views of COVID-19, in terms of what it is, how concerning it should be, what policies should be implemented, and what social obligations individuals should have in managing its spread. Although none of our questions and conversational probes addressed politics, interviewees spontaneously and repeatedly mentioned that the social and political atmosphere surrounding COVID-19 likely contributed to the public’s skepticism towards Long COVID.

Doubts about the impact of COVID-19 contributed to doubts about Long COVID. A death rate of 1% was perceived as low by the public even as medical and public health agencies regarded this figure as high [22,23]. Reports that certain groups (e.g., people who were elderly, obese, etc.) were disproportionately impacted might have led some to believe they were at no or negligible risk for COVID-19 and Long COVID [24]. Individuals who perceive that Long COVID is not a real or a significant medical condition would likely not volunteer for Long COVID research.

*Some people still don’t believe that it was a real thing, or [that it] was perpetrated by the government……. some people just want to say, oh, yeah, that wasn’t really a big deal. I don’t know why such a big deal was made out of that (6, 59, recovered)*

*So, if you are in bad health and you’re doing things bad, and people are saying, “Hey, this is what COVID attacks you. You know, this is how COVID hits you. It hits your immune systems of, you know, people that are over age or I’m sorry overweight, or you know, and as they say or maybe older people, that they have maybe lower immune systems, and you know, maybe they just… their bodies have probably maybe broken down a little bit more which can make some sense. So, like I feel like that maybe it’s just kind of run its course. (15, 62, recovered)*

Media reports about COVID appeared to have declined over time [25,26]. Other timely or more immediate concerns were prioritized. This in turn led to a lack of awareness, knowledge, and interest in Long COVID.

*Lack of awareness, yeah, or you know, more worried about I don’t know it’s the fall, and you got the flu. And with the RSV. And competing…news. She’s like I didn’t even know that there was like a long COVID syndrome. So yeah, I think it probably a lot of lack of awareness. (1, 28, recovered)*

Political opinions and commentary about the pandemic confused the public. Regardless of their political preferences, interviewees disliked the intermingling of politics with medicine and science as it made it harder for the public to discern what information was true and important to know.

*[O]bviously the country’s a little bit split up about their beliefs about COVID so I think you guys have an uphill battle with the political views, religious views and all those kind of things where it’s sometimes amazing to believe that that would get in the way of people to getting vaccination for example and getting treated and that’s why a lot of people died when it wasn’t necessary (10, 26–35 age range, recovered)*

*I think they they’re [referring to some politicians] so misinformed they’re trying to get people to do things …. Sir [addressing hypothetical politician] you are not a scientist you are not a doctor. chemist and nothing like that. Please be quiet keep your beliefs to yourself. (12, 65, recovered)*

*I believe… yes, it is real. I’m not denying that it’s not real. I mean, obviously people have died from it. But then, again, I could argue that it has become so political that it’s gone to the point where the bureaucrats have made it to where it… you can’t really draw line anymore to how the facts are in the reporting. (15, 62, recovered)*

Consequently, the discord surrounding COVID-19 and the polarization of views based on political allegiances might have led to reluctance to being diagnosed or linked with COVID-19 or Long COVID. If one’s preferred political party, social circle, and behaviors repudiated or downplayed the effects of SARS-CoV-2 infection previously, identifying publicly later as being affected or concerned about Long COVID would mean acknowledging one was wrong in the first place and/or could lead to social ostracization.

*Unfortunately, now, since the American political system has gone so out of hand, it’s such a joke, that with everything going on, you gotta basically walk on eggshells. So, I can see why people would not want their privacy being known, because God forbid that comes out. Maybe your employer feels the type of way about something. Maybe your girlfriend feels a type of way…. people don’t want to step on the wrong eggshell, and maybe I don’t know, you know, lack of better word, you know, nuke their, their, their… employment, career. (15, 62, recovered)*

*I know people that got it and they’re ashamed to say they had it too so if they could like scrub that out of their life they would. I know there’s a lot of people not comfortable saying they got COVID. Yeah because they were being irresponsible you know probably. (10, 26–35, recovered)*

*And I always used to think it was a fake disease. But now I seem to be experiencing it, and it hurts my heart. (8, 57, unrecovered)*

*COVID is still a super hot topic. So, when long lasting, Covid is mentioned, it could bring up some like emotions or thought processes that are negative. (7, 25, recovered)*

*Uncertainty surrounding institutions and COVID-19* represents the uncertainty interviewees express regarding the intentions, motivations, and positions of institutions involved with COVID. Greater uncertainty can lead to distrust and creates barriers to engagement. Suspicion existed about whether researchers and institutions might manipulate study participant data to support a pre-conceived, biased political agenda. Additionally, some believed that deliberately increasing the numbers of people affected by COVID would directly boost government funding for research institutions. Reservations were even extended to physicians and patient advocacy organizations.

*It depends, you know, because a lot of, the more you layers you peel from things, is it politically driven? Because I don’t want anything to do with politics, so I want something that deals more with science and real information, than to push a diagnosis because it politically fits somebody’s agenda. So if it’s a if it’s a citizen’s organization, I’d want to know more about it. I’d probably not participate because I don’t have trust in the citizens*. *(8, 59, unrecovered)*

*[I]f people are dying in hospitals… institutions or medical facilities are gaining tax dollar funded, or I should say, government funded programs. So, if you know, somebody dies and it’s from the COVID, they get a grants for whatever or somebody gets a positive Covid test, and you know they get some money, so it’s kind of a bureaucratic cash scheme in some aspects. Not all of it… But I mean, yeah, definitely money is a moving factor for it. (15, 62, recovered)*

*[I]f somebody reached out to me, and I just felt like it was a clear bias, you know, study where it says you just trying to obtain information that’s really only going to help your cause and not really get to the root of the problems. I guess what I’m trying to say is that, like I don’t like a double standard. So, if, if you’re looking for the truth, and you’re looking for honest opinions, and I feel that that’s what you’re seeking, then I’m willing to give my time to, you know, be one of the voices of either a few or maybe the many (15, 62, recovered)*

A secondary concern was whether researchers and research organizations had the knowledge, experience, and credentials to conduct meaningful research. Interviewees wanted their contributions to be used effectively and efficiently. Interviewees were particularly worried if the organization was one they were unfamiliar with or if it was a patient advocacy organization. Universities generally were viewed as having appropriate expertise.

*[A]s long as it’s an institution that I recognize, and I, you know. believe it. They’re doing the right thing. Then I would do it. (4, 59, unrecovered)*

*Uncertainty surrounding COVID-19 and COVID-19 research* refers to instances in which interviewees 1) express and/or discuss being confused or unaware as to what COVID and/or Long COVID are and 2) in which they communicate confusion and lack of awareness regarding COVID research in terms of what it is, what it seeks to accomplish, and/or how accurate it is. Interviewees believed they or the public possessed no or little knowledge and awareness about ongoing COVID research.

*Interviewer: What do you think of Long COVID research? Do you have any opinions?*

*Interviewee: No, I, I don’t know*

*Interviewer: Are you aware that the research is being conducted?*

*Interviewee: No, I pretty much felt that this COVID episode seems to have ran its course (15, 62, recovered*)

### Infringement

Infringement amplifies the normal, practical, day-to-day issues people must manage in order to participate in studies. The theme Infringement is composed of the subthemes *Invasiveness;* perceived *Lack of time;* and *Personal priorities, competing obligations, and the day-to-day.*

*Invasiveness* looks at how participation in COVID research might invade participants’ sense of privacy, impede their daily lives, and/or potentially introduce greater perceived risks into their lives. This includes financial burden (for example, missing hours from work) and “hassle.” Most respondents were satisfied with the privacy and confidentiality safeguards put in place for this and the overall COVID-UPP study, especially if they trusted the research group. Two respondents expressed concern initially about how they were targeted for recruitment into the study rather than about the information that would be collected. One of them, however, thought the issue was moot when so much personal data is available and shared amongst businesses and organizations.

*Yeah, I’m a little concerned. I mean, you guys did reach out to me at some point for the original study, and I guess that came through. I guess [health facility] somehow. so it concerns me a little bit. I guess that’s why I’m not turning my camera on too. (6, 59, recovered)*

Some people might prefer to take part in clinical trials, where they have the chance to try treatments, versus longitudinal, observational studies like COVID-UPP, meant to characterize epidemiology or understand COVID-19 pathophysiology. Thus, we asked interviewees whether study type might affect their involvement. Interviewees expressed that unknown side effects, pre-existing health issues that could be exacerbated, and a preference to avoid medications would make them hesitant to participate in studies of interventions.

*I already take a lot of pills more now since COVID than I ever have in my life, and I feel like maybe I’m over medicated. And I don’t want to take anything to make me feel worse. And if it’s experimental you know that actually frightens me. I don’t want to be part of something that will cause me to get cancer in 6 months, a year, or something, because it, you know. It was a good idea at the time, but not enough research was done for it. (8, 59, unrecovered)*

While some did not mind travelling for in-person or multiple study visits, others noted the extra time and energy they needed to expend to drive themselves or arrange to have someone drive them to on-site visits. One person did not have a car and another’s Long COVID cognitive symptoms prohibited them from driving. Interviewees noted that virtual videoconferencing and situating studies at sites they already frequented like their doctor’s clinic or workplace would help. If a study were of special interest to them or they felt their participation was vital, they explained they would be willing to put up with some inconveniences.

*[I]n person,…,my boyfriend had to take me to the clinic like that was really exhausting and challenging. The less I have to think [about transportation]..I could care less about the gift cards. (1A, 46, unrecovered)*

*[W]ell I think what you guys are doing right now with the zoom and it’s great, [Y]ou know this is way better than you know having to go places and report yourself. I think after COVID people’s lifestyle just really shifted into online stuff so I think what you guys are doing is the best way (10, 26–35, recovered)*

*Lack of time* identifies instances in which interviewees discussed time in relation to participating or not participating in COVID-19 research. Simple lack of time and schedules discordant with times studies were being conducted were also noted to be barriers.

*I can’t identify anything in in my life that it would make it difficult. Of course, like anything else is just aligning time with, you know, of course, time management with doing the research and scheduling. (9. 45, unrecovered)*

To overcome this barrier, interviewees suggested time-flexible participation opportunities (e.g., weekends and evenings), short questionnaires, and early information about the time estimated to participate. They also mentioned that if they were particularly interested in a study or felt their contributions were important, time might be less of a factor.

*[S]o I think that’s to me would be the warning you know? knowing what the time investment is, what does it look like? And describing it. (9, 45, unrecovered)*

*Personal priorities, competing obligations, and the day-to-day* involves how participation in Long COVID research may clash with an individual’s regular commitments. Difficulty accommodating research activities into one’s life diminishes the likelihood of participating. Family, work, and school obligations as well as recreational preferences were cited or affirmed by many interviewees as obstacles to participation.

*[M]y husband who is a Veteran and needs my help all the time. It is difficult for me to leave him alone. I also have work. (2A, 45, unrecovered)*

### Less effective means for engagement

These are outreach methods that do not prompt the public to participate in COVID research. Telephone calls, physical letters, and QR codes were not viewed as effective means of outreach. Most interviewees stated they would not answer telephone calls from an unrecognized person or number. They also paid less attention to unsolicited letters and noted that mail could be lost, delayed, or delivered to the wrong address. Several had heard of or seen QR codes before but did not necessarily know how to scan the code to obtain information. Two interviewees mentioned that older people might be less comfortable with newer technology.

### Trust

Trust assures individuals that their participation will not harm them, will be for a worthy cause, and will not unjustly benefit particular groups over others. Trust is composed of the subthemes *Familiarity and credibility of institutions involved with COVID-19*; *Appreciation, understanding, and respect for science and research*; and *Involvement of physicians in recruitment*.

*Familiarity and credibility of institutions involved with COVID-19* refers to the extent to which interviewees’ expressions of familiarity with institutions involved with COVID-19 and/or attributions of credibility to such institutions facilitates trust with the work those institutions do on COVID-19. Regardless of outreach method, interviewees stated they were more likely to pay attention to information and respond if they recognized the contacting individual or organization. Affiliation with a scientific or medically-oriented individual or organization, even if not direct (e.g., a friend, colleague, etc.) or close, also played a significant role: nine out of thirteen interviewees had been educated by, worked for, or knew someone affiliated with the sponsoring research or another health organization in some way. Rapport developed with research staff also made enrollment a positive experience for two interviewees.

*[Organization name] [is] a respected institution……My co-worker works for [component of organization]. Yeah. So I think that influenced me more [to participate in this interview] than if it was just some company that was just doing surveys or doing studies that I did. I wasn’t familiar with yeah. (6, 59, recovered)*

To increase participation, interviewees suggested that the name or logo of organizations be prominently displayed on public-facing materials. For organizations that they did not know, introduction of the study in a setting familiar to them (e.g., the clinic they attended) and more information about the researchers and study could help (e.g., participant tasks, organization accreditation).

*[I]f you’re going to be talking to soldiers, because you know, you get a VA stamp. And people the soldiers know that that’s a legitimate thing. Or you know your doctors, like your friendly doctor’s office, or you know, whatever it is that you can clearly see that, okay, this is a legit organization like I know this, I’ve seen this. This is recognizable, like this clearly is not a scam, you know, like basically anything that’s not going to show that this is scam. (15, 62, recovered)*

*Appreciation, understanding, and respect for science and research* refers to how interviewees’ knowledge and attitudes towards science and the process of scientific inquiry might affect their perception of Long COVID research and participation in Long COVID research. Interviewees viewed science and medical research as an overall positive and necessary means to find answers for COVID-19 and Long COVID. No one expressed negative views about the concept of science itself.

*Not that science has the answer or knows everything. But you know, with science we will get the answers. at least for the best of our ability. (6, 59, recovered)*

We specifically asked interviewees if changing or mixed messages about COVID-19 affected their views. Although one person stated messages could be confusing, several reported that they were not surprised by changing information. In fact, they recognized that change was a fundamental characteristic of science and anticipated our understanding would evolve. One person noted the whole situation had taught them to be more careful about the source of information when seeking answers.

*Well, you know that’s science. It’s all trial and error, it’s the scientific method. You try, you document it. You move on to the next thing until you see what works and what doesn’t…. to me it’s important that getting to the bottom of it, even though the diagnosis may change from time to time, and the outcomes. You know they’re trying. (8, 59, unrecovered)*

This subtheme was also linked with Personal Factors as individuals’ views on science are based on their past experiences with science.

*Physician involvement in recruitment* involves interviewees discussing whether they believed physicians are/ would be an effective means of recruiting folks to participate in Long COVID research and whether physicians should be used as a recruitment mechanism. Most interviewees felt that they would be more likely to participate in a study if their physician had brought it up. They trusted their own physician more than research groups and believed the doctor would not have initiated a discussion if the study did not fit their own individual health situation. Outside of personalized discussions, posters on clinic walls, flyers sent from their clinic, or physician co-signed recruitment materials were acceptable substitutes for their physician’s direct endorsement.

*[Physician suggestion would] make a difference…I will consider it seriously, because I trust my doctor…In fact, that’s how I ended up doing the study with the [organization] because, one of the doctors contacting me Say, look, you know you’d be a great candidate for this particular study.. (4, 59, unrecovered)*

*“the best thing is when the patient hears from his own doctor about the study*

*I think that this way the patient would feel much more confident and interested.*

*people would be much more motivated to participate in Long COVID studies” (2A, 45, unrecovered)*

### Administrative factors

Administrative factors can reduce friction in individuals’ decision-making processes when choosing to participate in studies. The theme administrative factors is composed of the subthemes *Participant-centeredness, Incentives,* and *More effective means for engagement*.

*Participant-centeredness* covers instances in which interviewees communicated ideas about how researchers could accommodate participants, rather than expecting the participants to accommodate the study. Adjusting certain aspects of a study can promote willingness to participate in COVID research. Scheduling study assessment times and travelling to any in-person visits were common problems for interviewees. Multiple interviewees recounted that if research appointments could be adapted to their schedule, they would be happy to participate in studies. Some did not have access to a car and others could not drive due to physical or cognitive symptoms so providing/ arranging for transportation might help. Alternatively, if study tasks could be performed around the time of a scheduled routine clinic visit, this would save them having to make an extra trip. Empathetic staff could also influence their decision. Different modalities of interactions – for example, written surveys, telephone calls, in-person visits – were also welcomed to accommodate the needs and preferences of interviewees.

*I take Uber right down to come out there it’s $33.66 that’s $60 round trip ….so I’m saying I can’t do this every time I go and luckily …my kids got cars they come pick me.*

*Have you ever like somebody come pick you up and take you there…. it’ll be probably the best thing yeah you probably get a lot of people there. Just make sure they can get there. (12, 65, recovered)*

*Incentives* cover the role of materials or services that are or could be offered to encourage participation in Long COVID research. Although interviewees appreciated tangible incentives such as the $20 Amazon gift card offered to speak to us, almost all cited it as a bonus and not the primary driving factor for their collaboration. When asked, most felt that the incentives offered were adequate and did not suggest another amount or type of incentive.

Of more value to them were health-related benefits, whether intended or not by researchers. Interviewees valued access to cutting-edge and extra information, specialized testing, and expert clinicians. Study appointments were regarded as additional clinical care.

*[M]y whole reason for doing any of this it’s self-preservation, self-interest. But I do love the gift cards. I do it, whether or not you sent me one, but I don’t want you to stop sending me. (8, 59, unrecovered)*

*I’m most interested because I want to see the lab results. That’s what really drove me to participate, and just to get some hopefully. Have some answers about this mysterious. This is what’s going on with me. (1A, 46, unrecovered) [participant who enrolled in COVID-UPP after enrolling in this study]*

*Participant: And of course, if you have any information, I’d love to know what will happen to me in the future in terms of the conditions.*

*Interviewer: So that you can get more care?*

*Participant: I mean I’m being seen [by the research staff] aren’t I? So yeah, that’s a type of care and again I’d appreciate any information you have. (3, 60, unclear recovery status)*

In contrast to themselves though, they felt the public needed such and even more inducements to consider taking part.

*To get more people to participate, you know you have to dangle a carrot…. the gift certificates are good… there’s people who won’t do anything unless they get paid. (8, 59, unrecovered)*

*More effective means for engagement* identifies approaches interviewees considered to be more persuasive in convincing them to participate in COVID research. Interviewees favored being contacted via e-mails or text messages as they checked their computers or mobile phones daily. These methods were also less disruptive to their lives: they could check and respond to messages whenever they wished. Multiple methods of communication were also suggested as interviewees noted individuals might differ in their preferences, reminders helped, and one method could reinforce another. Three interviewees also conveyed they would answer a phone call from a research group despite not recognizing the name/ number if they had been notified beforehand of a call.

### Personal factors

Personal factors can influence individuals’ degrees of willingness to participate in research. The theme personal factors is composed of the subthemes *Personal and social motivation, Personal experience with research*, and *Personal experience of COVID-19 and care*.

*Personal and societal motivation* captures interviewees’ actual and potential reasons for participating in Long COVID research. Finding out the cause of and treatments for Long COVID whether for themselves, their loved ones, or society was the top reason interviewees described as their motivation.

*COVID, you know it’s personal to me and my family, and it was a difficult time for everybody. and you know I’d like to help if there’s I know there’s people that are still suffering. and you know, if I can be a little small part of you know, helping that out. Why not? (6. 59, recovered)*

*I think as long as a study is going on I will participate in it and so forth. like I said I, I I’m thinking of the greater good for others as well as myself. (4, 59, unrecovered)*

Other reasons included relieving boredom; having the chance to learn more about Long COVID; assuring their views and those of people like them were heard; believing their contribution would have an immediate, meaningful impact; and volunteering due to having recruited patients as a researcher. Two interviewees suggested that people who were functionally impacted by Long COVID-19 might be more interested in participation.

*I’ve not signed up for studies at this university, because there wasn’t an impact that I could see directly… with this study I could see an impact that the information, the guidance I provide on what I would want from research. (5, 18–25, recovered)*

*Personal experience with research* communicates the extent to which interviewees, and/or folks within their family or social networks, have any prior experience of participating in studies or performing research. Five out of 13 interviewees had been subjects in a past or current research project (not necessarily COVID-related) and three knew someone who was a research subject. Experiences with research were described as positive or neutral.

*Personal experience of COVID-19 and care* reveals interviewees’ discussions of what it was like for them, their family, or their social networks to be sick with COVID. Since all our interviewees tested positive for SARS-CoV-2 at one point and about half were still affected, it was not surprising to hear they were participating out of self-interest.

Several participants expressed that obtaining medical care could be difficult, whether due to the lack of Long COVID-knowledgeable health care providers, effective treatments, or insurance coverage of possible treatments. For the vast majority, these challenging circumstances did not affect their participation in research. One interviewee mentioned how callous care made them less interested in participating initially.

Another interviewee recounted that cognitive symptoms and fatigue made participation difficult, but most did not feel that their symptoms limited their participation in this or the COVID-UPP study.

*Well. I have pain, I have fatigue, but it this is the extent of it. This is not going to keep me from participating. This is easy. I’m sitting down at a desk. I’m doing my job while I’m talking to you at the same time. It’s not a big deal (8, 59, unrecovered)*

### Unifying model

A unifying theme (Fig 1) emerging from our analysis of the 13 interviews suggests that within the context of recruiting participants for Long COVID studies, skepticism and uncertainty further complicate the process of individuals feeling comfortable to and choosing to participate in research. Skepticism, less effective means of engagement, and infringement experienced on the part of individuals being recruited for Long COVID research increase barriers to participation (Fig 2). Trust, administrative factors, and personal factors play into individuals’ willingness to participate in Long COVID research (Fig 3).

Whereas researchers can do little about individuals’ personal factors, they can address the themes of trust and administrative factors while reducing skepticism and infringement. Recruitment and study efforts that are participant-centered, respond to participant needs and preferences, and demonstrate tangible value can facilitate participation. Recruitment efforts that are informative, transparent, and engaging can clarify issues, establish rapport with recipients, and demonstrate intrinsic value to them, thus diminishing resistance to participation.

### Frequency analyses of data

Table 1 lists the number of text segments associated with each of the 15 subthemes and whether those text segments mentioned the subtheme as a facilitator, barrier, neither facilitator nor barrier, or unrelated to participation in research. For 30% or more of the times they were mentioned, the factors *Participant-centeredness, Personal and social motivation, Incentives*, and *Familiarity and credibility of institutions* were linked favorably with partaking in research. In contrast, when *Personal priorities and obligations, Lack of time, Social and political context,* and *Invasiveness* were touched upon, interviewees believed they inhibited participation.

**Table 1:** Segments of text classified by subthemes and sentiment.

Subtheme name	Total number segments	Facilitator segments n (%)^a^	Barrier segments n (%)	Neither Barrier nor Facilitator segments n (%)	Unclassified segments n (%)
**Appreciation, understanding, and respect for science and research**	42	8 (19)	5 (12)	3 (7.1)	26 (62)
**Familiarity and credibility of institutions involved with COVID-19**	82	29 (35)	6 (7)	11 (13)	36 (44)
**Incentives**	71	34 (48)	1 (1)	10 (14)	26 (37)
**Invasiveness**	84	21 (25)	35 (42)	15 (18)	13 (16)
**Participant-centeredness**	32	19 (59)	8 (25)	1 (3)	4 (13)
**Personal and societal motivation**	65	36 (55)	7 (11)	8 (12)	14 (22)
**Personal experience of COVID-19 and care**	146	4 (3)	4 (3)	2 (1)	136 (93)
**Personal experience of recruitment**	55	5 (9)	6 (11)	1 (2)	43 (78)
**Personal experience with research**	38	4 (11)	1 (3)	2 (5)	31 (82)
**Personal priorities, competing obligations, and the day-to-day**	25	2 (8)	18 (72)	3 (12)	2 (8)
**Physician involvement in recruitment**	48	12 (25)	1 (2)	0 (0)	35 (73)
**Social and political context**	62	10 (16)	27 (43)	2 (3)	23 (37)
**Lack of time**	41	7 (17)	21 (51)	8 (19)	5 (12)
**Uncertainty surrounding COVID-19 and COVID-19 research**	75	8 (11)	11 (15)	3 (4)	53 (71)
**Uncertainty surrounding institutions and COVID-19**	70	9 (13)	20 (29)	4 (6)	37 (53)

The first column shows the total number of text segments by subtheme. Subsequent columns subclassify text segments by sentiment: whether the text segment mentions the subtheme as a facilitator, barrier, or neither facilitator nor barrier to study participation. Unclassified segments describe text without a specific sentiment.

^a^Percentages are calculated by dividing the n of a specific column by the total number of text segments classified to that theme. For example, 34 out of 71 segments of text (or 48%) mentioned “Incentives” as a facilitator of participation. For some themes, the percentages add up to more than 100 as some segments of text included the theme as both a facilitator and barrier of participation.

Viewed from the perspective of facilitators and barriers, the top five most-mentioned facilitators were *Personal and social motivation; Incentives; Familiarity, and credibility of institutions involved with COVID-19; Invasiveness (lack of);* and *Participant-centeredness*. The top five most-mentioned barriers were *Invasiveness; Social and political context; Lack of time; Uncertainty surrounding institutions and COVID-19;* and *Personal priorities, competing obligations, and the day-to-day.* When this analysis was repeated examining how many and what percentage of interviewees indicated each subtheme as a facilitating, obstructing, or neutral factors (S4 Table), these subthemes once again emerged as the ones most mentioned by participants.

A comparison of barriers and facilitators between those interviewees who deemed themselves as recovered from SARS-CoV-2 infection and those who judged themselves to be unrecovered found these same subthemes ranked among the top five for each group, albeit the order of their ranking differed slightly (S5 Table). The biggest difference was seen with *Social and political context*: it was the top obstacle to participation for recovered interviewees but was the fifth-ranking obstacle for unrecovered interviewees.

**Fig 4:**
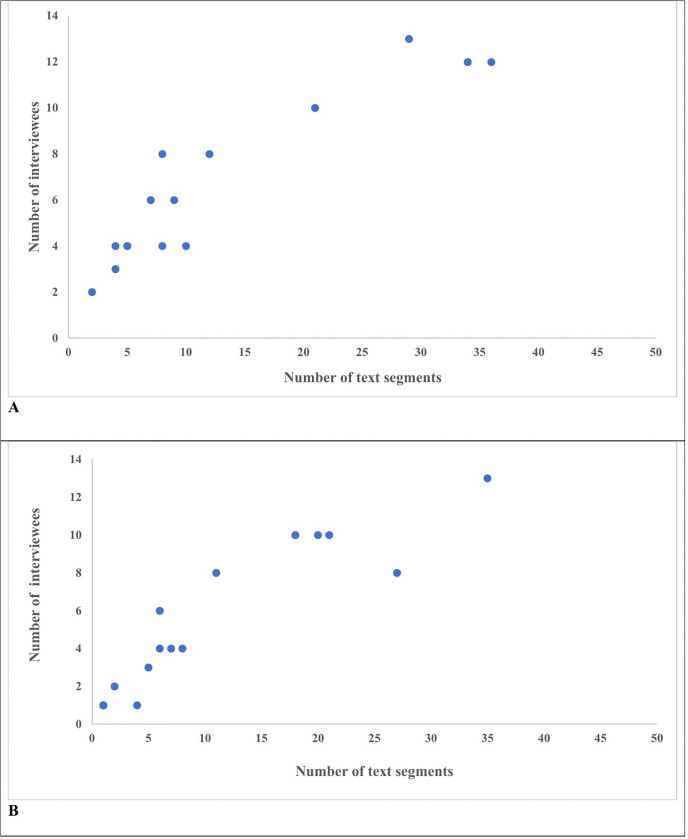
Number of text segments for a subtheme versus number of interviewees mentioning that subtheme. Each point represents a specific subtheme. The x-axis represents the overall number of text segments coded to that subtheme with that sentiment (facilitator or barrier) and the y-axis. the number of interviewees mentioning that subtheme with that sentiment. For example, if an interviewee mentions a subtheme ten times during their interview, the number of text segments is ten, but the interviewee is only counted once. **Panel A** refers to text segments coded as Facilitators and **Panel B;** text segments coded as Barriers.

For each sentiment (facilitator or barrier), the number of text segments classified under a subtheme was plotted against the number of interviewees (out of thirteen total) mentioning that factor (Fig 4). The scatterplots revealed a linear relationship with no factors in the upper left-hand or lower-right hand corner, suggesting that our interviewees agreed upon the role of the factors. That is, no factor was mentioned frequently by only a few interviewees or mentioned rarely but by a considerable number of interviewees.

## Discussion

This qualitative study of 13 individuals who declined participation in a Long COVID study describes relevant barriers and facilitators for participating in research. Among the top barriers and facilitators identified were the subthemes *Personal and social motivation, Incentives, and Social and political context* which appeared to exist independently from other issues. In contrast, other top barriers and facilitators represented the two ends of the spectrum of the same subtheme. For example, *Familiarity and credibility of institutions* and *Uncertainty surrounding institutions related to COVID-19* reflect the trust or lack of trust of research institutions and the individuals affiliated with them. In similar fashion, *Invasiveness, Lack of time, Participant-centeredness, and Personal priorities, competing obligations, and the day-to-day* all pertained to logistical aspects of participation that could be barriers or facilitators depending on the situation. Hence, we categorized the decision to participate in Long COVID research into five general but interacting themes: Skepticism, Infringement, Trust, Administrative Factors, and Personal Factors. Trust is the opposite of Skepticism while Administrative Factors can involve decreasing Infringement on people’s lives.

These themes and subthemes we found echo those that have been identified in the setting of clinical trial recruitment. In two separate reviews [13,27] and one qualitative data synthesis [28] examining factors affecting participation, common barriers included lack of time, logistical challenges, mistrust, and individual aspects of studies (e.g., randomization, risk or benefit of interventions) whereas common facilitators included altruism, ability to contribute meaningfully to science and medicine, personal benefits, and incentives.

Altruism consisted of both pure and conditional altruism, meaning the study participant felt they or a close contact would benefit from the research project, albeit not necessarily directly or immediately. Personal benefits in the setting of a clinical trial usually meant access to expensive or experimental treatments but study participants in our and other studies also highlighted access to information, testing, monitoring, and physicians as important to them. Incentives such as gift certificates or cash were viewed as bonuses by all but not central to volunteering. Two subthemes, trial information and external influences (e.g., family, friends, personal physician, media) could serve as either facilitators or barriers, depending on the content or person, respectively.

Although the purpose, tasks, and outcomes of epidemiologic studies like COVID-UPP differ from clinical trials, Slegers et al. [29] confirmed that altruism also played a key role in individuals’ decisions to participate in non-interventional research. They also reported that trust of researchers and their intentions by potential participants was the paramount factor underlying participation. Strȍmmer et al. agreed [30], hypothesizing that without a critical level of trust achieved with participants, other efforts to increase recruitment would be ineffective. For COVID-19 specifically, Barre et al. [14], found that among Black Americans, despite generally positive attitudes about research and concerns about COVID-19, long-standing distrust of scientist and scientific institutions was the strongest barrier to volunteering.

Against this backdrop, events and actions occurring during the pandemic eroded existing levels of trust. In the decades since 1980, 75% or more of Americans polled have viewed science as a positive force [31]. Confidence in scientists exceeded that for elected officials, religious figures, journalists, and business leaders. From January 2019 to January 2022 however, the percentage of people losing confidence in science increased significantly, jumping from 13% to 22% [32].

Subsequently, Long COVID inherited the confusion and skepticism originating with COVID. We noted that while our conversations with study participants were framed as concerning Long COVID, interviewees did not perceive acute COVID-19 as a separate entity from Long COVID but rather as one continual process. In contrast, medical and scientific professionals have focused on distinguishing between the acute and chronic effects of SARS-CoV-2 in their endeavors to understand and treat the latter [33,34]. Furthermore, although our interviewees did not express disbelief about Long COVID or lack of trust in institutions directly, they referred to others as being skeptical about Long COVID and mentioned multiple times their concerns about the intentions of scientists, physicians, and patient advocacy groups. In particular, interviewees who identified themselves as recovered brought up questions about the prevalence, severity, and relevance to society of LC. It is possible that some participants, seeking to avoid confrontation with us, chose to mask their own skeptical thoughts by transferring them to other parties. Recognizing the vulnerability our participants might feel, we took their comments at face value as, whether about themselves or others, they illuminate the overall atmosphere surrounding LC. Many had a positive view of science and even anticipated the science would change as the understanding of SARS-CoV-2 advanced. But they did not want to be part of any study driven by pre-conceived, profit-driven, or political agendas. Interviewees told us people affected by Long COVID might refuse the diagnosis due to guilt over rejecting preventive actions, conflict with their other strongly-held beliefs (e.g., if COVID-19 is not a serious infection, how can I be sick with Long COVID?), or fear of ostracization by their social circle. Thus, this group would likely not be interested in any Long COVID study.

What can be done then to increase recruitment under such circumstances? In their review of interventions to improve recruitment for clinical trials, Treweek et al. [35] found that a variety of techniques had been investigated but that the evidence was weak with replication studies hardly existent. Thus, they recommended only two techniques: making clinical trials open (rather than blinded and randomized) and following up unanswered physical letters with telephone calls. Later, Houghton et al. [28] and Sheridan et al. [27], independently, reviewed Treweek’s paper through the lens of barriers and facilitators and came to the same conclusions: a) tested interventions did not incorporate present knowledge of how people decide to be volunteers and b) interventions proposed in the future must do this to be effective,

In our unifying model, the theme Personal Factors and subthemes such as *Social and political context, Appreciation of science*, and *Personal experience of COVID-19 and care* are to a large degree not modifiable by researchers. Researchers cannot change an individual’s past cumulative experiences, media reports, political environment, or the social milieu individuals immerse themselves in. But they can take steps to address the subthemes Trust and Administrative Factors as well as components of their counterparts Skepticism and Infringement. In the following section, we describe possible ways to address these themes and subthemes.

### Build trust to decrease skepticism

The first strategy is to use trusted messengers to communicate research opportunities. Several interviewees pointed out they were familiar with the researchers’ organizations and thus felt comfortable volunteering. Local universities and medical groups could produce public content validating the existence of Long COVID and the need for research. Others suggested they would be more likely to volunteer if their physicians had spoken to them about the studies. Most believed that their personal physician had their best interests at heart and would not mention a study if there were no medical benefits for them. In lieu of direct discussions, information flyers in clinics or physician-endorsed materials could serve as proxies of physician approval. Frequently, regulatory bodies discourage this practice due to the possibility of coercion. However, there is increasing evidence that provider involvement can be a key to recruitment if processes are put in place to protect participants [36]. Community-based participatory research approaches are another option [37]. During the second half of 2022, COVID-UPP research staff began partnering with local LC patient advocacy groups to send out study invitations. This action led to an increase in response rates.

Another strategy is to demonstrate traits that make up what the public considers trustworthiness. During 2022, in reaction to misinformation and fake news, the Ad Council Research Institute [38] conducted a study to determine whom Americans trusted and why they trusted them. Physicians and scientists ranked highly, only behind spouses and relatives, as the parties people relied on to help them comprehend and to make decisions surrounding medical and scientific issues. Trustworthiness, as defined by over two-thirds of respondents, included possessing a history of honesty, sharing consistent information, avoiding conflicts of interest, being unbiased and independent, and presenting multiple points of view.

Combining these results with our interviewees’ comments and borrowing ideas from the field of marketing [39] yields multiple ways to gain trust. To establish credibility, a publicly oriented webpage could detail the researchers’ education, training, record of successfully completed projects, and other credentials. Disclosure of funders and statements that funders will not interfere with study conduct can allay concerns about conflict of interest [27,28]. Plain language explanations about the medical condition being researched can serve as evidence of the researchers’ knowledge. Testimonials from study participants may provide information from a participant-centered perspective [14,27] and function as approval from an ostensibly unbiased third party. Access to materials about what to consider before volunteering can decrease decision fatigue, demonstrate openness, and communicate researchers’ concerns for the general well-being of potential participants. To facilitate public understanding of the research process, the US Office for Human Research Protections has created a web-based button – linking to educational materials – that researchers can easily add to their websites [40].

For studies using medical databases to select participants, informing those contacted about how and why they are being contacted is important [28]. Before care is given, many patients must sign a consent form for treatment. Many of these forms include a clause telling patients their de-identified data may be used for research or asking if they wish to be contacted for medical studies. Since these forms can be long and dense, many people do not read them thoroughly or forget what they have signed. Therefore, some of our interviewees were caught off-guard when contacted as they were not sure how their names ended up on our outreach list. Two interviewees pointed out that this generated suspicion from them initially. In these circumstances, potential participants should be reminded of the forms they signed.

### Educate the public early about the medical condition being studied

Based on how recovered interviewees ranked *“Social and political context”* as their top barrier to participation, it appears that these individuals may be more influenced by or attuned to external, non-scientific sources (e.g., mainstream media outlets, social acquaintances) than individuals who are sick and thus are aware of COVID-19’s long-term impact through their lived experiences. Researchers should consider including brief, accurate information about the prevalence, severity, and negative consequences (e.g., job loss, inability to care for family) of the medical condition being studied in study recruitment materials. This may help healthy participants to understand why their involvement is needed, especially when misinformation about the medical condition is widespread, or recruitment of healthy participants has been difficult.

### Offer incentives valued by potential participants

Monetary rewards and gift certificates, although welcomed, were not the major drivers of participation. Instead, health-related benefits, the opportunity to help themselves and others, and the chance to advance science prompted people to sign up for studies.

Even without the possibility of treatment, study participants clearly recounted that additional information, testing, monitoring, and assessments by healthcare professionals would be valuable to them. Stating such study components as benefits might appear to conflict with the Belmont Report [41] and regulations governing research in the United States. They might be classified as “undue influence,” defined as “an offer of an excessive, unwarranted, inappropriate, or improper reward or other overture to obtain compliance,” especially for people who cannot afford healthcare. Such offers might overwhelm participants’ thinking process so much that risks are not adequately thought through leading to questionable informed consent [42]. Evaluating whether a benefit counts as an undue influence though is not straightforward and must be judged within the context of a specific study by institutional review boards.

However, authorities are starting to realize that allowing participants access to their own results respects the rights of and maximizes benefit for the participant. In 2018, the US National Academy of Medicine (NAM) published “Return of Individual-Specific Research Results Generated in Research Laboratories to “reviews [sic] the current evidence on the benefits, harms, and costs of returning individual research results” and to guide research stakeholders about if, when, and how results should be shared [43]. The report noted that a) researchers were already mandated to share with participants results which could affect their clinical care and b) since 2014, under the Health Insurance Portability and Accountability Act (HIPAA), participants already had the right to inspect their own results originating from HIPAA-covered laboratories. Consequently, researchers can appropriately and ethically state that some test results will be transmitted to participants.

To alleviate undue influence, the NAM Committee suggested that the benefits of receiving results, especially tests still under development, be portrayed accurately. If a test has not been validated or its clinical significance proven, those characteristics need to be divulged. With regard to other health-related benefits, testimonials might be one way to relay them. This technique is exemplified by the videos displayed on the “Building Trust Between Minorities and Researchers” website [44] where interviewees talk about why they participate in clinical trials. Of course, participants’ reasons should not be coerced but be given freely.

Emphasizing upfront the benefits associated with altruism can be complicated and if carried out incorrectly could instigate guilt. Nevertheless, recognition of participants’ altruism through personalized Thank You cards or via the acknowledgement sections of public presentations and papers might make participants feel appreciated. Newsletters and presentations explaining the progress, results, or consequences of a study can show volunteers the impact of their participation.

### Design participant-centered studies to minimize infringement on peoples’ lives

Interviewees believed that volunteering for research would infringe on their freedom of movement, energy, time, and schedules. Researchers can decrease travel for participants by conducting assessments virtually or travelling to places participants already frequent, like their homes, workplaces, or medical clinics. Alternatively, assistance with transportation to sites would be welcomed. Minimizing the length, difficulty, or time needed for tasks would be helpful. Our interviewees also liked being forewarned about how much time a task would take or how many more tasks did they needed to complete during an assessment session (e.g., via a progress bar). Finally, permitting research appointments to be scheduled in the evenings or on weekends would better accommodate many people’s lives.

For this study, multiple participants mentioned they appreciated the opportunity to participate virtually from anywhere, early information about how long the interviews would take, and the availability of interview times in the evening.

## Strengths

To our knowledge, this is the first study to examine factors influencing participation in Long COVID studies. By speaking to a diverse group of individuals who had been recently invited to participate in a Long COVID study but declined engagement at various stages of recruitment, we were able to transcend the theoretical and explore barriers and facilitators that motivate eligible individuals to participate or not participate in a Long COVID study. Since South Florida is a demographically and politically diverse area, the influence of conflicting messaging and political/ social context on research participation emerged clearly and could be explored. For many medical conditions, the surrounding political and social atmosphere may not affect research participation much. This is not the case for COVID-19 or Long COVID, where most of our interviewees specifically identified the political atmosphere as an influential factor. We hope the results of our study can inform recruitment for studies concerning politically controversial medical conditions.

## Limitations

Limitations of this study include interviewees being confined to those who were already engaged with a healthcare system, did not fully participate in COVID-UPP, and volunteered for the study. Because COVID-UPP was carried out with the cooperation of six healthcare systems, we did not reach out to people receiving care from other healthcare systems or who could not access any formal health care. These individuals might report or prioritize a different set of facilitators and barriers. Including people who enrolled in COVID-UPP might have offered a different perspective and allowed us to compare motivations and obstacles between those who did and did not participate in COVID-UPP. People with Long COVID-associated cognitive challenges might not have been able to sustain a 30-minute conversation while those with extreme views against participation might not have volunteered to be interviewed. The thoughts and opinions expressed by our sample based in one region of South Florida might not reflect those of a larger group or other areas of the United States. Nevertheless, we believe our interviews uncovered several factors relevant to those conducting Long COVID research.

Our total study population of 13 participants is small. Qualitative studies like this one are designed to illustrate possible views on the topic of interest, rather than to be representative of the views of a population, as a quantitative study would. No new themes arose in this study after the tenth participant’s interview. In qualitative research, saturation, or the point at which data become repetitive and no new insights are emerging through interviewing additional subjects, is a criterion for stopping data collection. The sample size of 13 in this study is within the range of the number of interviewees (9–17) that Hennink et al., through a systematic review, found necessary to reach saturation [45].

## Future directions

Studies evaluating the public’s current knowledge, attitudes, or behaviors towards Long COVID could help illuminate factors to consider when recruiting for Long COVID studies. Recent surveys from the US Centers for Disease Control and Prevention and the Pew Research Center found, respectively, that between twenty and thirty percent of Americans had not heard of Long COVID [46,47]. Most of the literature examining barriers and facilitators has focused on recruitment into clinical trials. Yet studies of etiology, pathophysiology, diagnosis, and prognosis which do not involve treatment interventions are also important and the reasons why the public might participate in such studies might differ from clinical trials. Research pertaining to the factors affecting participation in observational studies would be helpful. Finally, we agree with Sheridan et al. [27] and Houghton et al. [28] that randomized controlled trials of recruitment interventions directed at identified factors, like those we and they have observed, are greatly needed.

## Conclusions

This study sought to identify participant-related factors influencing participation in Long COVID research. Some facilitators and barriers are like those recognized previously. Unique to COVID-19 and Long COVID, we found that the social and political context of the pandemic contributed to distrust or skepticism towards research organizations and their employees. Thus, establishing and promoting trust such as through utilizing trusted messengers or community-based participatory research efforts is especially vital. Removing logistical barriers, sharing accurate medical information, and offering incentives participants value might also improve recruitment rates. Given the millions of people globally who are affected by Long COVID and the heavy burden this disease imposes on their lives, development of effective recruitment strategies is crucial to finding solutions for them.

## Supporting information

S1 File Interview Guide. (DOCX)

S2 File COREQ Checklist. (PDF)

S1 Table Barriers to participation: definitions of themes and subthemes. (DOCX)

S2 Table Facilitators of participation: definitions of themes and subthemes. (DOCX)

S3 Table Coding tree with themes, subthemes, and open codes. (DOCX)

S4 Table Subthemes and sentiment by number and percentage of interviewees out of 13 interviewees. (DOCX)

S5 Table Top five facilitator and barrier subthemes ranked by number of text segments and classified by self-reported health status of interviewees. (DOCX)

## References

[1] Davis HE, McCorkell L, Vogel JM, Topol EJ. Long COVID: major findings, mechanisms and recommendations. Nat Rev Microbiol. 2023;21(3):133–46. doi: 10.1038/s41579-022-00846-2 36639608 PMC9839201

[2] National Center for Health Statistics, US Census Bureau. Long COVID - Household Pulse Survey - COVID-19. 2024 March 21 [cited 2024 April 5]. In: US Center for Disease Control and Prevention [Internet]. Available from: https://www.cdc.gov/nchs/covid19/pulse/long-covid.htm

[3] Bach K. New data shows long Covid is keeping as many as 4 million people out of work. In: Brookings [Internet]. 24 Aug 2022 [cited 2024 Apr 8]. Available from: https://www.brookings.edu/articles/new-data-shows-long-covid-is-keeping-as-many-as-4-million-people-out-of-work/

[4] Tziolos NR, Ioannou P, Baliou S, Kofteridis DP. Long COVID-19 pathophysiology: what do we know so far?. Microorganisms. 2023;11:2458. doi: 10.3390/microorganisms11102458 37894116 PMC10609046

[5] Greenhalgh T, Sivan M, Perlowski A, Nikolich JŽ. Long COVID: a clinical update. The Lancet. 2024;404:707–24. doi: 10.1016/S0140-6736(24)01136-X 39096925

[6] Ely EW, Brown LM, Fineberg HV. Long Covid Defined. N Engl J Med. 2024;391:1746–53. doi: 10.1056/NEJMsb2408466 39083764 PMC11687645

[7] RECOVER Initiative. A year of discovery: looking back at 2025 and ahead to 2026 | RECOVER COVID Initiative. In: Recovercovid.org [Internet]. 2026 Jan 13 [cited 2026 Feb 19]. Available from: https://recovercovid.org/news/year-discovery-looking-back-2025-and-ahead-2026

[8] RECOVER Initiative. Studies | RECOVER COVID Initiative. In: Recovercovid.org [Internet]. [cited 2026 Feb 19]. Available from: https://recovercovid.org/studies

[9] Hamlin RE, Blish CA. Challenges and opportunities in long COVID research. Immunity. 2024;57(6):1195–214. doi: 10.1016/j.immuni.2024.05.010 38865966 PMC11210969

[10] Cohrs R. After nine months, an update on NIH’s long Covid research. In: STAT [Internet]. 2022 Dec 22 [cited 2024 Feb 7]. Available from: https://www.statnews.com/2022/12/22/after-nine-months-an-update-on-nihs-long-covid-research/

[11] Thoelke K. There’s a silent crisis in clinical research. And it’s not Covid-19. In: STAT [Internet]. 2020 Oct 28 [cited 2024 Feb 7]. Available from: https://www.statnews.com/2020/10/28/recruitment-retention-silent-crises-clinical-trials/

[12] Briel M, Olu KK, von Elm E, Kasenda B, Alturki R, Agarwal A, et al. A systematic review of discontinued trials suggested that most reasons for recruitment failure were preventable. J Clin Epidemiol. 2016;80:8–15. doi: 10.1016/j.jclinepi.2016.07.016 27498376

[13] Rodríguez-Torres E, González-Pérez MM, Díaz-Pérez C. Barriers and facilitators to the participation of subjects in clinical trials: An overview of reviews. Contemp Clin Trials Commun. 2021;23:100829. doi: 10.1016/j.conctc.2021.100829 34401599 PMC8358641

[14] Barre I, Cunningham-Erves J, Moss J, Parham I, Alexander LR, Davis J. Motivators and Barriers to COVID-19 Research Participation at the Onset of the COVID-19 Pandemic in Black Communities in the USA: an Exploratory Study. J Racial Ethn Health Disparities. 2023;10(6):2890–9. doi: 10.1007/s40615-022-01466-5 36512311 PMC9746576

[15] Charmaz K. Grounded Theory: Objectivist and Constructivist Methods. In: Lincoln NKD a YS, editor. Handbook of Qualitative Research. 2nd ed. Thousand Oaks, CA: Sage. 2000.

[16] Strauss A, Corbin J. Basics of qualitative research: techniques and procedures for developing grounded theory. 2nd ed. Thousand Oaks, CA: Sage. 1998.

[17] McConnell‐Henry T, Chapman Y, Francis K. Husserl and Heidegger: Exploring the disparity. Int J of Nursing Practice. 2009;15:7–15. doi: 10.1111/j.1440-172X.2008.01724.x 19187164

[18] Braun V, Clarke V. Using thematic analysis in psychology. Qualitative Research in Psychology. 2006;3(2):77–101. doi: 10.1191/1478088706qp063oa

[19] Charmaz K. The grounded theory method: an explication and interpretation. In: Glaser B, editor. More grounded theory: a reader. 2nd ed. Mill Valley, CA: Sociology Press. 1994.

[20] Glaser B, Strauss A. The discovery of grounded theory: strategies for qualitative research. 2nd ed. New York: Aldine de Gruyter. 1999.

[21] Bryant A, Charmaz K. The SAGE handbook of grounded theory. Los Angeles: SAGE. 2007.

[22] Coltrain N. Fact check: Does COVID-19 have a mortality rate of 1%-2%? USA TODAY. 2020 May 5 [cited 2026 Feb 19]. Available from: https://www.usatoday.com/story/news/factcheck/2020/05/05/covid-19-fact-check-coronavirus-mortality-rate-misleading/3019503001/

[23] Vijay SL. Less Than 1% Of All Infected Individuals May Die From COVID-19, But Easy Transmissibility Makes The Virus Dangerous, Says WHO - Health Policy Watch. In: Health Policy Watch [Internet]. 2020 Aug 3 [cited 2026 Feb 27]. Available from: https://healthpolicy-watch.news/less-than-1-of-all-infected-individuals-may-die-from-covid-19-but-easy-transmissibility-makes-the-virus-dangerous-says-who/

[24] Caldera C. Fact check: Low body fat, healthy lifestyle do not prevent COVID-19. USA TODAY. 2020 July 30 [cited 11 Jun 2024]. Available from: https://www.usatoday.com/story/news/factcheck/2020/07/30/fact-check-low-body-fat-healthy-lifestyle-do-not-prevent-covid-19/5516078002/

[25] Joseph K, Horne BD, Green J, Wihbey JP. Local news online and COVID in the U.S.: relationships among coverage, cases, deaths, and audience. ICWSM. 2022;16: 441–52. doi: 10.1609/icwsm.v16i1.1930

[26] Ali SMA, Sherman-Morris K. Pandemic and health reporting: A content analysis of New York times coverage of COVID-19 from January 01, 2020, to August 31, 2022. Social Sciences & Humanities Open. 2023;8:100739. doi: 10.1016/j.ssaho.2023.100739

[27] Sheridan R, Martin-Kerry J, Hudson J, Parker A, Bower P, Knapp P. Why do patients take part in research? An overview of systematic reviews of psychosocial barriers and facilitators. Trials. 2020;21(1):259. doi: 10.1186/s13063-020-4197-3 32164790 PMC7069042

[28] Houghton C, Dowling M, Meskell P, Hunter A, Gardner H, Conway A, et al. Factors that impact on recruitment to randomised trials in health care: a qualitative evidence synthesis. Cochrane Database Syst Rev. 2020;10(10):MR000045. doi: 10.1002/14651858.MR000045.pub2 33026107 PMC8078544

[29] Slegers C, Zion D, Glass D, Kelsall H, Fritschi L, Brown N, et al. Why Do People Participate in Epidemiological Research?. Bioethical Inquiry. 2015;12(2):227–37. doi: 10.1007/s11673-015-9611-2 25672617

[30] Strömmer S, Lawrence W, Rose T, Vogel C, Watson D, Bottell JN, et al. Improving recruitment to clinical trials during pregnancy: A mixed methods investigation. Soc Sci Med. 2018;200:73–82. doi: 10.1016/j.socscimed.2018.01.014 29421474 PMC6033317

[31] Funk C. Key findings about Americans’ confidence in science and their views on scientists’ role in society. In: Pew Research Center [Internet]. 2020 Feb 12 [cited 7 Feb 2024]. Available from: https://www.pewresearch.org/short-reads/2020/02/12/key-findings-about-americans-confidence-in-science-and-their-views-on-scientists-role-in-society/

[32] Funk BK Alec Tyson and Cary. Americans’ Trust in Scientists, Other Groups Declines. In: Pew Research Center Science & Society [Internet]. 2022 Feb 15 [cited 2024 Feb 7]. Available from: https://www.pewresearch.org/science/2022/02/15/americans-trust-in-scientists-other-groups-declines/

[33] Perego E, Callard F, Stras L, Melville-Jóhannesson B, Pope R, Alwan NA. Why the Patient-Made Term “Long Covid” is needed. Wellcome Open Res. 2020;5:224. doi: 10.12688/wellcomeopenres.16307.1

[34] National Academies of Sciences, Engineering, and Medicine. Long-term health effects of COVID-19: disability and function following SARS-CoV-2 infection. Washington, DC: The National Academies Press. 2024. doi: 10.17226/27756 39312610

[35] Treweek S, Pitkethly M, Cook J, Fraser C, Mitchell E, Sullivan F, et al. Strategies to improve recruitment to randomised trials. Cochrane Database Syst Rev. 2018;2(2):MR000013. doi: 10.1002/14651858.MR000013.pub6 29468635 PMC7078793

[36] Wilson S, Draper H, Ives J. Ethical issues regarding recruitment to research studies within the primary care consultation. Fam Pract. 2008;25(6):456–61. doi: 10.1093/fampra/cmn076 18953068

[37] Christopher S, Watts V, McCormick AKHG, Young S. Building and maintaining trust in a community-based participatory research partnership. Am J Public Health. 2008;98(8):1398–406. doi: 10.2105/AJPH.2007.125757 18556605 PMC2446462

[38] Ad Council Research Institute. 2022 Trusted Messengers Study. In: Ad Council Org [Internet]. 2022 [cited 2024 Apr 8]. Available from: https://www.adcouncil.org/learn-with-us/ad-council-research-institute/2022-trusted-messengers-study/full-report-form

[39] Brudner E. 11 Psychology Tips to Get Prospects to Trust You Faster. In: Hubspot [Internet]. 2021 June 15 [cited 2024 Apr 8]. Available from: https://blog.hubspot.com/sales/psychology-tips-that-will-get-your-prospects-to-trust-you-faster

[40] United States Department of Health and Human Services Office for Human Research Protections. Get a Web Button From OHRP That Links to the About Research Participation Website! 2019 Nov 6 [cited 2024 Apr 8]. In: US Department of Health and Human Services [Internet]. Available from: https://www.hhs.gov/ohrp/education-and-outreach/about-research-participation/get-a-web-button/index.html

[41] United States Department of Health and Human Services Office for Human Research Protections. Protections. Read the Belmont Report. 2018 Jan 15 [cited 2024 Apr 8]. In: US Department of Health and Human Services [Internet].Available from: https://www.hhs.gov/ohrp/regulations-and-policy/belmont-report/read-the-belmont-report/index.html

[42] Hosely M. The Many Faces of “Coercion” and “Undue Influence.” 2020 Dec 2 [cited 2024 Feb 11]. In: Advarra [Internet]. Available from: https://www.advarra.com/blog/the-many-faces-of-coercion-and-undue-influence

[43] Botkin JR, Mancher M, Busta ER, Downey AS. Returning individual research results to participants: guidance for a new research paradigm. Washington, D.C.: National Academies Press. 2018. doi: 10.17226/25094 30001048

[44] University of Maryland Center for Health Equity. Should I Participate? | Building Trust - Between Minorities and Researchers. [cited 2024 Apr 8]. Available from: http://buildingtrustumd.org/unit/informed-decision-making/should-i-participate

[45] Hennink M, Kaiser BN. Sample sizes for saturation in qualitative research: A systematic review of empirical tests. Soc Sci Med. 2022;292:114523. doi: 10.1016/j.socscimed.2021.114523 34785096

[46] Long COVID - NCHS Rapid Surveys System. 2024 Feb 22 [cited 2024 May 14]. Available from: https://www.cdc.gov/nchs/rss/round1/long-covid.html

[47] Pasquini AT and G. How Americans View the Coronavirus, COVID-19 Vaccines Amid Declining Levels of Concern. 2024 Mar 7 [cited 2024 Apr 8]. In: Pew Research Center Science & Society [Internet].Available from: https://www.pewresearch.org/science/2024/03/07/how-americans-view-the-coronavirus-covid-19-vaccines-amid-declining-levels-of-concern/

