# Supplementary material for: Facilitators of and barriers to participation in Long COVID research: A qualitative analysis
Source: PLoS One. 2026 May 6;21(5):e0346007. doi: 10.1371/journal.pone.0346007 (PMC13148652; doi:10.1371/journal.pone.0346007)
Supplement: S1 File — (DOCX) [file pone.0346007.s001.docx]

**S1 File: Interview Guide**

Hi, my name is _______________. I am a member of the COVID-UPP Qual Research team and will be conducting your interview today.

Thank you for taking the time to help us understand how people decide to take part In LC studies. If this call is disconnected, please click the ZOOM link again in your invitation. If you still cannot connect, call us at _______________ and we will help you.

I will be asking you a series of questions. The interview will last approximately 30 minutes, but it could be up to an hour if you wish to share more, we have up to 1 hour. You can stop the interview at any time.

**Housekeeping**

There are no right or wrong answers to these questions. I am interested in your personal experiences, so feel free to share your own situation, thoughts, and opinions. I want to hear everything that you have to say. If you don’t feel comfortable answering a question, just let me know and we can skip it.

There also might be pauses between questions. I want to give you time to answer the questions. It will also help me take notes.

As was mentioned before, this interview will be recorded. I don’t want to miss anything you say. We want to capture your exact wording. Do you feel comfortable with recording our discussion?

A window will appear telling you when recording has started. Please click to confirm you are fine with recording.

[START RECORDER]

We already asked this before, but I’d like to get it on record. Can you confirm that you would like to take part in this project?

Opening questions

**Some people who had COVID-19 can experience long-term effects after their infection, known as post-COVID or long COVID conditions. Long COVID conditions include a wide range of health problems that can last weeks, months, or longer.**

1. **Has your COVID diagnosis affected your life? If yes, how?"**

**If “Yes, worse than before diagnosis....",**

Have you sought care for your symptoms? How do you think your care has been? Are you

feeling better, worse, or the same now?

PROBES

- Do you think it is easy or difficult for people to get the care they need after COVID? Why?
- How do you think your experience has been compared to others who live with symptoms which remain after COVID?

**If “No, not worse”:**

[For non-LC] Do you know anyone who has been affected by LC? How do you think they have been treated? What do you think about the health care that people with long COVID conditions are getting?

1. **Is this your first time taking part in a study?**

- **If “NO,”** “What study did you take part in before? What did you think of it? Would you do it again? Why or why not? Did that experience affect your decision to take part today?
- **If “YES,”** “Has anyone you know ever taken part in a study? Did their experiences have any effect on your decision to take part today?

1. **What do you think of long COVID research?**

**Does the subject of the research affect your decision to take part?**

PROBE TOPICS

- Awareness of medical/ LC research
- Attitudes about medical research in general
- Attitudes about LC medical research
- Abundance of COVID research
- Impact of research on people with long COVID conditions
- Influence of media on perceptions of COVID research
- **Personal and/or family/ friend experiences with research**
- Where do you get your information about LC studies?
- Compared to the people around you, do you consider yourself just as, more, or less aware of LC research?

Barriers to taking part in research

**I am so glad [name], you are willing to talk to me today. Thanks again for participating in this study. Now, I’m going to ask you some questions about what might make you decide to take part in a study.**

1. **What made you decide to take part in the interview today?**

PROBE TOPICS

- Learning more about long COVID
- Feeling like they are being cared for
- Sharing experiences to help others
- Legitimacy
- Incentive
- Communication about the project

1. **What are some reasons that you would decide not to take part in any/ the COVID-UPP research study?**

**PROBE TOPICS - ask specifically if not brought up by interviewee**

- Health issues - fatigue, pain, problems thinking, low energy (esp. LC group)
- Concerns about privacy, confidentiality, way results will be used
- Logistics - comfort with technology, driving/ parking, time of interview
- Competing tasks - work, school, family care
- Incentives - not worth the time, effort
- Communication about the project

1. **Would the type of study affect whether you take part or not? For example, studies testing a drug vs. those just checking on your health? Studies that only require 1 visit vs. 2+ visits vs. virtual visits (i.e., can be done anytime, anywhere like this interview)?**
2. **How could we make it easier or more attractive for people [with long COVID conditions] to participate in research? What type/ amount of incentive would be reasonable for this study?**

Trust in project sponsors

1. **During the last 2 years, information about COVID-19 and long COVID has often changed. Sometimes, new information is the opposite of old information, leading to a lot of confusion. That has led some people to no longer trust scientists or physicians. What do you think or how do you feel about this situation? How do you feel about taking part in a study run by Nova Southeastern University (NSU)? …the CDC? patient organizations? …your doctor?**

**PROBE TOPICS**

- Unaware of institutions
- Level of trust

Recruitment and survey mode preferences

**Your answers have been very helpful. The next group of questions are about how you would.**

**like to be informed about studies.**

1. **You were contacted by e-mail initially. Was this a good way to reach you? Did you receive it in your Inbox? What attracted you to the e-mail? Sender? Subject line?**

PROBE TOPICS

- Legitimacy of information?
- What emails rise to the top of significance/importance?
- How important is the title of the email when responding?

1. **What is the best way to contact you for a research study—in-person, by phone, email, text,**

**“snail” mail (letter)—and why?**

PROBE TOPICS

- Phone, letter, email, text
- Frequency of use
- Comfort/ease of use

1. **What is the worst way to contact you for a research study and why?**

PROBE TOPICS

- Phone, letter, email, text
- Concerns about legitimacy or privacy

1. **Are you familiar with QR codes?** **QR codes are made up of black and white squares and can be read by your phone camera to direct you to a webpage. What do you think about using a QR code to get information about a COVID study?**

PROBE TOPICS

- Familiarity with QR codes
- Security/legitimacy concerns
- Comfort with technology

1. **As part of this study, you had to complete a short survey online first. How did you feel about that? What do you think about completing a survey online vs. other ways (e.g., phone, paper)?**

PROBE TOPICS

- Access and comfort in completing online survey
- Confusing, uncomfortable, difficult to answer questions (why/ where stop?)
- Security concerns

Closing question

We’re coming to the end of the interview. Just one more question…

1. The main goal of this project is to understand why some people may not take part in long COVID studies. Is there anything else you would like to share that can help me better understand some of the concerns?
