# Supplementary material for: Facilitators of and barriers to participation in Long COVID research: A qualitative analysis
Source: PLoS One. 2026 May 6;21(5):e0346007. doi: 10.1371/journal.pone.0346007 (PMC13148652; doi:10.1371/journal.pone.0346007)
Supplement: S1 Table — (DOCX) [file pone.0346007.s003.docx]

| ***Barriers to participation: themes, subthemes, and definitions*** | |
| --- | --- |
| ***Theme: Skepticism* diminishes individuals’ willingness to participate, comfort to participate,**  **and/or sense of confidence and safety in participating.** | |
| ***Skepticism subthemes*** | ***Subtheme definition*** |
| Social and political context | *Social and political context* captures interviewees’ expressions of how they perceived others’, and their own, political and cultural views of and approaches to COVID-19, in terms of what it is, how concerning it should be, what should be done about it in terms of policies, and what social obligations individuals should or should not have in managing the spread of COVID-19. |
| Uncertainty surrounding institutions and COVID-19 | *Uncertainty surrounding institutions and COVID-19* represents the extent to which interviewees express uncertainty regarding the intentions, motivations, and positions of institutions involved with COVID. |
| Uncertainty surrounding COVID-19 and research | *Uncertainty surrounding COVID-19 and research* refers to instances in which interviewees 1) express and/or discuss being confused or unaware as to what COVID and/or long COVID are and 2) in which they communicate confusion and lack of awareness regarding COVID research in terms of what it is, what it seeks to accomplish, and/or how accurate it is. |

| ***Theme: Infringement* amplifies the normal, practical, day-to-day issues people must manage**  **in order to participate in studies** | |
| --- | --- |
| ***Infringement subthemes*** | ***Subtheme definition*** |
| Invasiveness | *Invasiveness* looks at how interviewees express the extent to which participation in COVID research invades their senses of privacy, impedes their daily lives, and/or potentially introduces greater perceived risks into their lives. This includes financial burden (for example, missing hours from work) and “hassle.” |
|  |  |
| Personal priorities, competing obligations, and the day-to-day | *Personal priorities, competing obligations, and the day-to-day* involves interviewees expressing how opportunities to participate in, and activities of participating in, COVID-19 research run up against a constellation of other commitments, personal and/or family priorities, work, and general management of one’s day-to-day goings-on. The extent to which one’s priorities, obligations, and day-to-day things to get done do not, or cannot, accommodate participation in COVID-19 research diminishes one’s likelihood of participating. |
| Lack of time | *Time* identifies instances in which interviewees discuss time in relation to participating or not participating in COVID-19 research. |
| ***Theme: Less effective means for engagement* identifies instances in which interviewees discuss approaches that are less effective in engaging them to consider participating in COVID-19 research.** | |

**Supplemental Table 1.** Barriers to participation: definitions of themes and subthemes.
