# Supplementary material for: Facilitators of and barriers to participation in Long COVID research: A qualitative analysis
Source: PLoS One. 2026 May 6;21(5):e0346007. doi: 10.1371/journal.pone.0346007 (PMC13148652; doi:10.1371/journal.pone.0346007)
Supplement: S2 Table — (DOCX) [file pone.0346007.s004.docx]

| ***Facilitators of participation: Themes, subthemes, and definitions*** | |
| --- | --- |
| ***Theme: Trust* facilitates confidence among individuals that their participation in long COVID research**  **will not harm them, serve a specific purpose, and be for a good cause.** | |
| ***Trust subthemes*** | ***Subtheme definitions*** |
| Familiarity and credibility of institutions involved with COVID-19 | *Familiarity and credibility of institutions involved with COVID-19* refers to the extent to which interviewees’ expressions of familiarity with institutions involved with COVID-19 and/or attributions of credibility to such institutions facilitates trust with the work those institutions do on COVID-19 |
| Physician involvement in recruitment | *Physician involvement in recruitment* involves interviewees discussing whether or not they believe physicians are/ would be an effective means of recruiting folks to participate in COVID-19 research and whether or not physicians should be used as a recruitment mechanism. |
| Appreciation, understanding, and respect for science and research | *Appreciation, understanding, and respect for science and research* refers to how the extent to which interviewees understand, appreciate, and respect science and the process of scientific inquiry influences the interviewees’ perception of COVID-19, COVID-19 responses, COVID-19 research, and potential consideration of participating in COVID-19 research. |

| ***Theme: Administrative factors* have the ability to reduce friction in**  **individuals’ decision-making processes for choosing to participate in studies.** | |
| --- | --- |
| ***Administrative factor subthemes*** | ***Subtheme definitions*** |
| Participant-centeredness | *Participant-centeredness* covers instances in which interviewees communicate ideas about the extent to which the study works to accommodate participants, rather than expecting the participants to accommodate the study. |
| Incentives | *Incentives* covers interviewees’ discussions of the role of incentives in enticing them to participate in COVID research and the types of tangible, concrete incentives offered, or that could be offered, to facilitate participation in COVID research |
| More effective means of engagement | *More effective means for engagement* identifies instances in which interviewees discuss approaches that are more effective in engaging them to consider participating in COVID research.  *Includes hypothetical and perceptions of issues affecting others* |
| ***Theme: Personal factors* influence individuals’ degrees of willingness to participate in research in ways that**  **are not particularly amendable by researchers but that can nevertheless encourage participation in research.** | |
| ***Personal factors subthemes*** | ***Subtheme definitions*** |
| Personal and societal motivation | *Personal and societal motivation* captures interviewees’ declarations of why they decided, or why they might decide, to participate in COVID-19 research in terms of what motivates them to do so, whether it be personal interest and/or an interest in doing good and helping others.  *This also includes interviewees’ perceptions of others’ motivations, keeping in mind the same exclusions* |
| Personal experience of COVID-19 and care | *Personal experience of COVID-19 and COVID-19 care* reveals interviewees’ discussions of what it was like for them, and/or folks within their family or social networks, to experience being sick with COVID-19, recovering from COVID-19, and/or the COVID-19 care they did or did not receive. |
| Appreciation, understanding, and respect for science and research | See above. |
| Personal experience with research | *Personal experience with research* communicates the extent to which interviewees, and/or folks within their family or social networks, have any prior experience of participating in studies or performing research |

**Supplemental Table 2.** Facilitators of participation: definitions of themes and subthemes.
