# Supplementary material for: Facilitators of and barriers to participation in Long COVID research: A qualitative analysis
Source: PLoS One. 2026 May 6;21(5):e0346007. doi: 10.1371/journal.pone.0346007 (PMC13148652; doi:10.1371/journal.pone.0346007)
Supplement: S3 Table — (DOCX) [file pone.0346007.s005.docx]

| **Themes** | **Focused Codes** | **Open Codes** |
| --- | --- | --- |
| Skepticism | Social and political context | Social context surrounding COVID-19 |
|  |  | Public views |
|  | Uncertainty surrounding institutions and COVID-19 | Uncertainty surrounding institutions and COVID-19  (Open code that was raised to the analytical level of a focused code) |
|  | Uncertainty surrounding COVID-19 and COVID-19 research | Uncertainty surrounding COVID-19 and COVID-19 research  (Open code that was raised to the analytical level of a focused code) |
| Infringement | Invasiveness | Invasiveness |
|  |  | Required to be physically present |
|  |  | Treatment vs. non-treatment study |
|  |  | Privacy |
|  | Time | Time and scheduling |
|  | Personal priorities, competing obligations, and the day-to-day | Priorities and the day-to-day |
|  |  | Competing obligations |
|  |  | Distracted by something else |
|  |  | White noise |
| Trust | Familiarity and credibility of institutions involved with COVID-19 | Familiar party |
|  |  | Institutional familiarity and credibility |
|  | Appreciation, understanding, and respect for science and research | Value and importance of science and research |
|  |  | Scientific process |
|  | Involvement of physicians in recruitment | Relevance of physicians for engagement |

| Administrative Factors | Participant-centeredness | Participant-centeredness  (Open code that was raised to the analytical level of a focused code) |
| --- | --- | --- |
|  | Incentives | Medical incentive |
|  |  | Non-medical incentive |
|  |  | Role of incentives |
|  | More effective means for engagement | There were 11 different “modality” open codes. Instances wherein respondents suggested a particular modality would be a more effective means for engaging them to consider participating in COVID research compose the focused code, “more effective means for engagement.” |
| Personal Factors | Personal and social motivation | Personal and societal motivation |
|  |  | Desire to help |
|  |  | Advance science |
|  |  | Meaningful contribution |
|  | Personal experience with research | Personal experience with research |
|  |  | Prior personal research experience |
|  |  | Others' prior research experiences |
|  |  | COVID-19/Long COVID research |
|  | Personal experience of COVID-19 and care | Personal context surrounding COVID-19 |
|  |  | Long COVID medical Care |
|  |  | COVID-19/Long COVID illness |
|  |  | Personally affected (including family) |
|  |  | Experience of Long COVID |

**Supplemental Table 3.** Coding tree with themes, subthemes, and open codes.
