# Supplementary material for: Facilitators of and barriers to participation in Long COVID research: A qualitative analysis
Source: PLoS One. 2026 May 6;21(5):e0346007. doi: 10.1371/journal.pone.0346007 (PMC13148652; doi:10.1371/journal.pone.0346007)
Supplement: S4 Table — (DOCX) [file pone.0346007.s006.docx]

|  | **Overall** | | **Facilitators** | | **Barriers** | | **Neutral** | |
| --- | --- | --- | --- | --- | --- | --- | --- | --- |
| **Subtheme** | **Number of Responders** | **Percentage of Responders** | **Number of Responders*** | **Percentage of Responders** | **Number of responders** | **Percentage of Responders** | **Number of Responders** | **Percentage of Responders** |
| Appreciation, understanding, and respect for science and research | 13 | 100.0% | 8 | 61.5% | 3 | 23.1% | 3 | 23.1% |
| Familiarity and credibility of institutions involved with COVID | 13 | 100.0% | 13 | 100.0% | 4 | 30.8% | 8 | 61.5% |
| Incentives | 12 | 92.3% | 12 | 92.3% | 1 | 7.7% | 6 | 46.2% |
| Invasiveness | 13 | 100.0% | 10 | 76.9% | 13 | 100.0% | 10 | 76.9% |
| Participant-centeredness | 7 | 53.8% | 7 | 53.8% | 4 | 30.8% | 1 | 7.7% |
| Personal and societal motivation | 12 | 92.3% | 12 | 92.3% | 4 | 30.8% | 6 | 46.2% |
| Personal experience of COVID and COVID care | 13 | 100.0% | 3 | 23.1% | 1 | 7.7% | 2 | 15.4% |
| Personal experience with research | 12 | 92.3% | 4 | 30.8% | 1 | 7.7% | 1 | 7.7% |
| Personal priorities, competing obligations, and the day-to-day | 10 | 76.9% | 2 | 15.4% | 10 | 76.9% | 3 | 23.1% |
| Relevance of physicians for engagement | 8 | 61.5% | 8 | 61.5% | 1 | 7.7% | 0 | 0.0% |
| Social context and public views of COVID | 10 | 76.9% | 4 | 30.8% | 8 | 61.5% | 2 | 15.4% |
| Time | 11 | 84.6% | 6 | 46.2% | 10 | 76.9% | 3 | 23.1% |
| Uncertainty surrounding COVID and COVID research | 13 | 100.0% | 4 | 30.8% | 8 | 61.5% | 2 | 15.4% |
| Uncertainty surrounding institutions and COVID | 12 | 92.3% | 6 | 46.2% | 10 | 76.9% | 4 | 30.8% |

^*^ Interviewees may mention a subtheme as a facilitator, barrier, or neutral factor at separate times during their interview.

**Supplemental Table 3.** Subthemes and sentiment by number and percentage of interviewees out of 13 interviewees.
