# Supplementary material for: Facilitators of and barriers to participation in Long COVID research: A qualitative analysis
Source: PLoS One. 2026 May 6;21(5):e0346007. doi: 10.1371/journal.pone.0346007 (PMC13148652; doi:10.1371/journal.pone.0346007)
Supplement: S5 Table — (DOCX) [file pone.0346007.s007.docx]

**Recovered Interviewees** **Unrecovered Interviewees**

| **Facilitator Subthemes** | **Number of**  **text segments** |  | **Facilitator Subthemes** | **Number of**  **text segments** |
| --- | --- | --- | --- | --- |
| Incentives | 16 |  | Incentives | 16 |
| Personal and societal motivation | 14 |  | Familiarity and credibility of institutions involved with COVID | 15 |
| Familiarity and credibility of institutions involved with COVID | 13 |  | Personal and societal motivation | 15 |
| Invasiveness | 9 |  | Participant-centeredness | 14 |
| Relevance of physicians for engagement | 6 |  | Invasiveness | 10 |

| **Barrier Subthemes** | **Number of**  **text segments** |  | **Barrier Subthemes** | **Number of**  **text segments** |
| --- | --- | --- | --- | --- |
| Social and political context | 20 |  | Invasiveness | 18 |
| Invasiveness | 14 |  | Time | 10 |
| Uncertainty surrounding institutions and COVID | 14 |  | Personal priorities, competing obligations, and the day-to-day | 8 |
| Time | 11 |  | Uncertainty surrounding institutions and COVID | 6 |
| Personal priorities, competing obligations, and the day-to-day | 9 |  | Social and political context | 6 |

**Supplemental Table 4.** Top five facilitator and barrier subthemes ranked by number of text segments and classified by self-reported health status of interviewee.
